# Life Space and Activity Space Measurement: Making “Room” for Structural Racism

**DOI:** 10.1093/geront/gnad160

**Published:** 2023-11-28

**Authors:** Sarah L Szanton, Kamila A Alexander, Boeun Kim, Qiwei Li, Gilbert C Gee, Karen J Bandeen-Roche, Paris B Adkins-Jackson, Melissa D Hladek, Laura J Samuel, Emily A Haozous, Safiyyah M Okoye, Deidra C Crews, Roland J Thorpe

**Affiliations:** School of Nursing, Johns Hopkins University, Baltimore, Maryland, USA; School of Nursing, Johns Hopkins University, Baltimore, Maryland, USA; School of Nursing, Johns Hopkins University, Baltimore, Maryland, USA; College of Health and Human Services, California State University, Fresno, California, USA; Department of Community Health Sciences, Fielding School of Public Health, University of California, Los Angeles, California, USA; Department of Biostatistics, Bloomberg School of Public Health, Johns Hopkins University, Baltimore, Maryland, USA; Departments of Epidemiology and Sociomedical Sciences, Mailman School of Public Health, Columbia University, New York, New York, USA; School of Nursing, Johns Hopkins University, Baltimore, Maryland, USA; School of Nursing, Johns Hopkins University, Baltimore, Maryland, USA; Southwest Center, Pacific Institute for Research and Evaluation, Albuquerque, New Mexico, USA; College of Nursing and Health Professions, Drexel University, Philadelphia, Pennsylvania, USA; Division of Nephrology, Department of Medicine, School of Medicine, Johns Hopkins University, Baltimore, Maryland, USA; Johns Hopkins Alzheimer’s Disease Resource Center for Minority Aging Research, Bloomberg School of Public Health, Johns Hopkins University, Baltimore, Maryland, USA

**Keywords:** Aging in place, Disparities (health, racial), Diversity and ethnicity, Function/mobility, Social networks

## Abstract

As we age, the ability to move is foundational to health. Life space is one measure of a person’s ability to move and engage in activity beyond the home. A separate but related concept is activity space, a measurement of a person’s spatial behaviors and visited locations that include social networks, neighborhoods, and institutions. In this article, we integrate the literature on life space and activity space, discussing how physical function is not only determined by individual capabilities, but also by the surrounding social and environmental factors, which may limit their agency. We show how structural racism contributes to inequities within this paradigm linking related concepts of movement, agency, belonging, and timing. We also explore implications for research and theory for mobility, social connection, and activity.

The ability for older adults to move within the context of their environments is vital to their functional independence. Gerontologists tend to focus on older adults’ limitations in mobility, such as decreased gait speed, inability to walk a quarter of a mile, or climb a flight of stairs, all of which consistently predict adverse health outcomes (Davis et al., 2015; Musich et al., 2018; Wilby, 2019). There is less focus on what older adults *can* do and *actually do.* Although measures of mobility limitation assess potential restrictions in movement, they do not illuminate how older adults truly use their everyday environments (Chung et al., 2015; Peel et al., 2005). There are two similar but distinct concepts that explore relationships between mobility in an environment and broader health outcomes: life space and activity space. In this article, we compare and contrast both concepts, describe how inequities and structural racism affect each, and contextualize each concept to discern the meaning and sense of belonging that spaces provide. We use the following definition of structural racism, “the totality of ways in which societies foster racial discrimination through mutually reinforcing systems … . These patterns and practices in turn reinforce discriminatory beliefs, values, and distribution of resources” (Bailey et al., 2017, p. 1455; Krieger, 2014, p. 650). Finally, we discuss research and theoretical implications of each concept, including how they relate to and can be strengthened by situating structural racism across the life course of older adults.

## Life Space

*Life space* refers to the socioecological environment in which an individual lives. The term describes movements in one’s environment such as leaving the house or neighborhood (Bayat et al., 2021; Johnson et al., 2020). The concept of life space was first introduced by May and colleagues in 1985 to define the area that participants moved within a specific period of time (May et al., 1985). May et al. (1985) used a life-space diary to quantify movements of community-dwelling older adults at home and in the immediate home environment. Since then, the concept of life space has been extensively used for conceptualizing and assessing mobility in older adults (Webber et al., 2010). For instance, Tinetti and Ginter (1990) used the life space concept to measure the extent and frequency of mobility among nursing home residents (Tinetti & Ginter, 1990). Stalvey et al. (1999) developed the Life Space Questionnaire to cover a broader range of regions beyond the immediate home environment as community-dwelling older adults often travel beyond the immediate home environment. Another self-reported measure, the Life Space Assessment that captures not only the extent of movement in the environment but also frequency of travels and type of assistance needed (Allman et al., 2006; Baker et al., 2003), is the most widely used instrument (Johnson et al., 2020). An older adult’s restricted life space indicates lower degree of mobility in terms of size of the area in which they move, frequency of trips, or level of independence. Restrictive life space is predictive of a range of adverse health outcomes such as preventable hospitalizations, need for skilled nursing care, and life expectancy (Johnson et al., 2020; Kennedy et al., 2017; Sheets et al., 2021). Although studies on racial disparities in life space are limited, research shows that racially minoritized older adults have restricted life space, which is predicted by low education, diabetes, and dementia (Allman et al., 2004). Black older adults compared to their White peers were more likely to report life space that restricted them to their own neighborhoods (Choi et al., 2016). Disparities in health outcomes among racially minoritized older adults, including earlier onset of mobility limitation and steeper declines of mobility function when compared with non-Hispanic Whites, were documented (Thorpe et al., 2008).

## Activity Space

A related concept is *activity space*, defined as “locations and networks within which individuals have direct contact as a result of their day-to-day activities” (Golledge & Stimson, 1997, p. 279). In 1970, the term activity space appeared, which was first developed by behavioral geographers (Brown & Moore, 1970; Horton & Reynolds, 1970; Patterson & Farber, 2015). The concept has since evolved and is applied in multiple disciplines such as urban and transportation planning, epidemiology, and public health but has been rarely used in gerontology research (Bayat et al., 2021; Smith et al., 2019; Patterson & Farber, 2015). Cagney and colleagues (2013) further developed the activity space concept, defining it as the set of places where social networks, neighborhoods, and institutions overlap in a person’s daily life. Activity spaces capture meaning within sociospatial experiences that develop through dynamic movements across and within geographic locations, fundamentally influencing individual health (Cagney et al., 2020). Activity space itself, or social or physical environments within activity spaces, can be measured using multiple objective and subjective measures including Global Positioning System (GPS) or Geographic Information Systems combined with questionnaires or diaries designed to elicit self-reported activities (Wong & Shaw, 2011; Yi et al., 2019; Zenk et al., 2019). Conceptually, activity space has utility when examining primary spaces where individuals spend time outside the home (Bayat et al., 2021; Hirsch et al., 2014). From a practical perspective, activity spaces are holistic, interactive, and relational, and include health-related and environmental factors individuals encounter in their daily lives (Crawford et al., 2014; Golledge & Stimson, 1997; Mason et al., 2010; Sherman et al., 2005). For example, Browning et al. (2017) found that people living in the same neighborhood do not necessarily share the same activity spaces, leading to limited social interaction and integration. Size and location of activity spaces may differ for racial and ethnic groups. Silm and Ahas (2014) found that a minoritized ethnic group in Estonia had smaller activity spaces (i.e., visiting a smaller number of districts) than their peers and were more likely to visit places where their own ethnic group was concentrated. African American and Latino individuals in the United States tended to conduct their routine activities such as working or going grocery shopping in areas with greater concentrations of poverty, joblessness, female-headed families, and “secondary sector workers” and few professional occupations or college graduates compared to White people (Krivo et al., 2013). Senior centers and grocery stores might be more accessible (e.g., closer proximity, longer hours of operation, and wheelchair accessible) in less segregated communities compared to more segregated ones.

## Comparing Life Space and Activity Space

Life space and activity space have contrasting strengths in measuring capacity, social network, and agency (Table 1). Life space measures the geospatial range of usual activity and infers limitations are due to diminished physical capacity (Baker et al., 2003). Life space does not explicitly consider environmental limitations caused by policies that design inflexible and inequitable layouts of housing, transportation, community resources (i.e., stores and libraries), and neighborhoods. Because it is a self-reported measure of where a person goes, the effects of depression and specific diseases such as dementia, as well as housing, transportation, and other civic resources within neighborhoods are “baked in” to the answer but are not explicitly examined. Activity space, on the other hand, not only measures the social and built environments where individuals actually go but also attends to the people they encounter while there (Cagney et al., 2013). Individual preferences, values, and characteristics such as income, race, or physical capacity are implicitly reflected in where people visit, but activity space does not explicitly consider individual characteristics as variables affecting physical movement or engagement. Life space explicitly asks participants whether someone needs human help to move out of the home or out of the neighborhood (Baker et al., 2003), but does not assess who is involved in the relationship. In contrast, activity space includes concepts that influence the space and the activities taking place within it such as social network characteristics and quality of social connections (Alexandre et al., 2020; Mason et al., 2015), individual and structural agency (where people choose to conduct daily activities or characteristics chosen for them; Bell et al., 2014; Ivory et al., 2015), and behavioral engagements (what they do when they are there; Zhang et al., 2019). Social networks and activity spaces are related but not the same. For example, when someone goes to the Senior Center to play bridge, they are simultaneously moving to an activity space (the Senior Center) and engaging with their social networks (friends). However, there are activity spaces (e.g., grocery stores) that do not usually involve social networks.

**Table 1.** Strengths, Limitations and How Structural Racism is Reflected in Life Space and Activity Space

Concept	Definition	Strengths	Limitations	How Structural Racism Is Reflected in It
Life space	Geospatial movement with and without human or device help over a quantified period of time (such as 2 weeks)	Subjective measure, recently use objective measure like GPS tracking device Summary measure of function	Can be conflated with depression and dementia Does not consider the accessibility of the environment	Ability to function physically is partially determined by life-course history of structural racism; ability to function in a place is partially determined by person–environment fit, which is also partially determined by structural racism
Activity space	The locations where social networks, neighborhoods, and institutions overlap in a person’s life to fundamentally influence individual health	Objective measure, subjective measure can be used like a travel diary Summary measure of space used	Usually cross-sectional or measured over short time period Does not consider individual characteristics or capacity such as assistance required for movement	Separate activity spaces due to segregation and segregated opportunities Important intersections of racism and ableism which both limit accessibility

*Note*: GPS = Global Positioning System.

Life space and activity space overlap in their perspective on resources needed for movement and the condition of the environment in which a person is moving. In other words, one’s measurable life space (e.g., individual capacity/mobility) may shape activity space (e.g., neighborhoods, institutions, and networks) or vice versa. Therefore, the application of both concepts demonstrates synergies that provide unique insights on the health trajectories and quality of life among older adults. Both approaches to understanding human behavior and the relationship between aging, place, and movement are valuable in aging research. In a world of growing isolation, considering people along with places is increasingly important.

## Life Space and Activity Space: The Impact of Structural Racism

Limitations in life space and activity space are due to both individual (e.g., physical disability and cognitive decline) and environmental (e.g., lack of transportation and unwalkable neighborhoods; Cagney et al., 2013) factors. Early onset of mobility limitations and steeper decline of mobility function due to environmental factors may be a pathway through which structural racism contributes to poor health outcomes in minoritized older adults. Contemporary and historical policies like segregation, discriminatory city planning practices, and uneven resource allocation have direct links to individual experiences that ultimately limit movement. For example, although an older adult may have the physical ability to leave a house and travel to another area of their community, they might not choose to because of crumbling infrastructure or lack of transportation in disinvested parts of cities. On the other hand, centuries of federal policies steeped in structural racism have resulted in American Indian and Alaska Native communities that are drastically underresourced, with many reservations lacking in basic public services considered essential for day-to-day living. Poor-quality roads and few reliable public transportation options create tangible limitations to movement for many Native American Elders, particularly for those living in rural and frontier regions of the United States (Bennion et al., 2022; Sommerfeld et al., 2021). Even basic infrastructure considerations such as funding for animal control services on reservation lands create substantial limitations in movement for Native American Elders. A widespread problem of free-roaming dogs limits the ability to safely access outdoor space for social visits and exercise, contributing to isolation and cognitive decline (Cardona et al., 2023; Griffith, 2022; Smith, 2007; Rickert, 2023).

Life space and activity space implicitly reflect and measure systems of power that operate within geographic boundaries. Neighborhood resources, often clustered in tightly bounded communities and resource poor environments, detract from well-being (Gill et al., 2021; Padeiro et al., 2022). Examples are financial drivers that designate services to areas based on potential for profit rather than public health need (Dickman et al., 2017), political drivers that allocate resources to areas deemed preferable to local leadership (White et al., 2015), and social processes such as residential segregation (Gaskin et al., 2012). This geographic spacing can severely alter the ways in which older adults interact with members of their communities, seek help from formal institutions, and reinforce the isolation that many face. Yet, neither life space nor activity space measures examine whether people feel they belong in spaces. People may inhabit or visit spaces for purely utilitarian purposes, but never feel they are welcome in those spaces. Stigmatizing spaces likely contribute to weathering among racially minoritized people, contributing to health deterioration in early life as a result of the cumulative impact of chronic exposure to social and economic inequities and political marginalization (Geronimus, 1992; Geronimus et al., 2006; Simons et al., 2018; Williams, 2018). In this way, the benefit of activity space access may be lessened or amplified for marginalized people depending on their experience of this space. Structural racism has segregated racially minoritized people in all facets of life, both at the microlevel (e.g., seating in a restaurant) and the macrolevel (e.g., immigration policy). Segregation is enforced by violence at the individual level, practices of institutions, and policies enacted by the government (Gee & Hicken, 2021). Although legal segregation has been outlawed, the practice of segregated spaces persists. These practices include segregation in employment, residence, and education, among others, relegating people to different spaces (Williams & Collins, 2001).

Structural racism is related to life space and activity space through the domains of education, employment, income/credit/wealth, civic engagement, healthcare, neighborhood factors, policing, environment, and media/marketing (Table 2; LaFave et al., 2022). Although the domains are discrete categories, a person can experience overlapping inequities in multiple domains, with a likely cumulative effect over time (Gee & Ford, 2011). Exposure to these domains is uneven, with those who are most affected by structural racism (e.g., people racialized as Native Americans and Black, residents of severely disinvested neighborhoods) experiencing the highest impacts to life space and activity space as a result. In addition, these factors likely have a cumulative effect over time, shaping disparities that include mobility limitations over the life course (Gee et al., 2019).

**Table 2.** Structural Racism Domains, Indicators, and Consequences

Domains of Structural Racism	Relevant Factors That May Create Inequities in Life Space and/or Activity Space	Consequences on Life Space and/or Activity Space
Housing	• Household crowding • Housing quality • Housing accessibility	• Limited activities in the home environment • Difficulty maintaining independence in and around the home • Limited social engagement, both in and out of the home environment
Education	• Quantity of educational opportunities • Quality of education • Social capital accrued during training	• Limited opportunities for upward social mobility and employment • Less social capital
Employment	• Accessible workplace environments • Flexible work requirements (timing and place of work) • Personal agency/control and workplace demands • Interacts with healthcare with regard to health insurance access • Opportunities for workplace advancement	• Less control/agency over life space and/or activity space • Less flexibility for other nonwork daily activities (work–life balance) • Fewer employment opportunities for those with disabilities or caregiving responsibilities • Limited opportunities for future work-based activities
Earnings	• Inequities in average earnings based on race • Interacts with employment with regard to ability to negotiate pay	• Fewer opportunities for leisure activities (hobbies, vacations) • Restricts future work activities and options • Creates need to work as many years as physically possible
Benefits	• Inequities in opportunities and flexibility to apply for and use benefits	• Less flexibility in daily activities (paid leave, retirement options, healthcare options)
Credit	• Inequitable mortgage practices • Inequitable rental practices • Intergenerational transfer of wealth limiting education, housing. and work options	• Restricts residential neighborhoods • May limit housing quality and/or accessibility
Media	• Perpetuating stereotypes that contribute to racial profiling, interpersonal, and systemic racism • Inequitable representation at all levels of media industry	• Perpetuates interpersonal discrimination and trauma • Contributes to both physical safety and sense of belonging across places and activities
Healthcare	• Access to healthcare resources (primary care, mental health, pharmacies, etc.) • Unconscious bias and distrust • Interacts with employment with regard to health insurance access	• Increases the effort, time, and travel distance required to engage in health-promoting behaviors and health management • Limits options for places to seek healthcare
Criminal justice	• Sense of safety and belonging • Differential rates of incarceration • Interacts with employment with regard to getting jobs after incarceration	• Restricts activities in many daily activities • Restricts employment for those with incarceration history
Civic Participation	• Barriers to accessing voting locations • Difficulty with participating in civic processes due to working hours, transportation, and unfriendly environments	• Low influence in policymaking • Limited representation in lawmaking

## Implications

The concepts of life space and activity space underscore the need for understanding where people go, how and why they go there, and how opportunities to engage in those spaces lead to fuller participation in society. The domains of structural racism can be measured through places where people have lived, such as where they were born, educated, voted, worked and retired. Once researchers have the physical addresses of these places across the life course for individuals (LaFave et al., 2022; Szanton et al., 2022), they can build data sets of opportunity and restriction to better understand the trajectory of these inhabited spaces and resources. The literature currently focuses on more macrolevel constraints on life/activity spaces, as implied by research on neighborhood segregation or state-level discriminatory policies (Athey et al., 2021). However, it would be useful to also consider the more microlevel forces such as police–citizen interactions, teacher–student ratios in public schools, and other manifestations of racialized constraints to life and activity spaces (Wiley & Shiffman, 2012). We need research with geographical context that is not just about individual strengths and attributes but ,includes the sociopolitical background shaped by structural racism.

Evaluating which groups feel welcomed and included in different spaces may then be important for research on activity spaces and health. Both moderation and mediation models are relevant. Structural racism likely modifies the associations between spatial characteristics and health outcomes so that minoritized groups derive less benefit from otherwise positive activity spaces due to experiencing failures of belonging within them. Even if the size of life space or activity space is the same, the quality of the place such as accessibility of health-promoting resources and experiences in those places can be different across racial groups, in turn, lead to differential health impacts. A mediation pathway we hypothesize is that failure of belonging contributes to avoiding otherwise beneficial activity spaces, which in turn loses health benefits for minoritized older adults. For example, a recent study noted that Black persons living in segregated neighborhoods had greater decline in cognitive processing speed over time (Besser et al., 2023). The authors were not able to explain the reason for this decline, but perhaps it is mediated via activity spaces (segregation constrains activity spaces which then leads to cognitive decline). If future researchers include and contextualize structural racism and resilience in their studies (Szanton et al., 2022), the explanatory power of life space and activity space can better guide interventions. Inclusion of these concepts means that if Black older adults have smaller life space, for example, interventionists may need to prioritize structural interventions as well as individual-level interventions that increase engagement in activity spaces through intentional expansion or enhanced quality of social networks (e.g., daily activities include people they can trust).

There are multiple policy implications from the concepts of life space, activity space, and what is omitted from each. For example, society can make policy investments to achieve universal access to all neighborhoods and spaces no matter someone’s physical function, age, or race. These could include more universal access to transportation and to making the spaces feel welcoming. Policies that advocate for the importance of fostering social connections in spaces as standard public health practice can play an important role in fostering community resilience to reduce and eliminate health inequities driven by unequal distribution of resources across places. In addition, equitable access to high-quality healthcare, consistent broadband internet, door-to-door mobility transport, access to safe green space, and vibrant, well-funded senior centers as well as income to support engagement in activities could reduce racial disparities in life space and activity space because people would be able to access more spaces and have the health and income to do so. They could start to break down geographical limitations and support robust relationships while aging for all people.

The content of this paper has theoretical implications. The theories of life space and activity space do not currently account for effects of structural racism. The underpinnings of the theories assume that all people may have free movement, unrestricted by structural racism’s bounding of contemporary and historical experiences. Either through old laws, the current effects of previous laws (e.g., redlining), or the structured gradients of mobility across race, structural racism defines attributes of both life space and activity space. Rather than “controlling for” race in models using activity space and life space, it is important to operationalize structural racism to analyze its effects on mobility, activity, disability, and belonging among older adults in the spaces they inhabit. A number of studies have emerged showing ways to operationalize institutional, cultural, and structural racism (Adkins-Jackson et al., 2022; Dean & Thorpe, 2022; Groos et al., 2018; LaFave et al., 2022; Michaels et al., 2023). When these life space and activity space concepts include structural racism, they will be better able to measure the real experience of older adults in environment. Quantifying size of life space or activity space without consideration of the structural racism may miss the opportunity to understand underlying causes of size or characteristics of life or activity space, exposure and interaction to social and physical environments, and their impact on health.

## Conclusion

As a society, we gain if all people can inhabit any space that they need (McGhee, 2021). In gerontology, we have focused on older adults and their autonomy as though movement and mobility throughout space is an individual asset or deficit rather than examining sociopolitical effects such as structural racism on life space. Learning on activity space’s emphasis on social connection and freedom will be important in next phases of research and in policy-making.

## Data Availability

The authors do not report data because no data set was used to complete this work. Therefore, the preregistration and data availability requirements are not applicable.
